# The Perceptual and Aesthetic Aspects of the Music-Paintings Congruence

**DOI:** 10.3390/vision3040065

**Published:** 2019-11-20

**Authors:** Katarina Rančić, Slobodan Marković

**Affiliations:** Laboratory for Experimental Psychology, University of Belgrade, 11000 Belgrade, Serbia; katarinarancic@yahoo.com

**Keywords:** abstract paintings, music, aesthetic preference, congruence, regularity, complexity

## Abstract

The purpose of the present study is to investigate the effect of congruence between music and paintings on the aesthetic preference of paintings. Congruence was specified as the similarity in perceived regularity and the complexity of jazz compositions and abstract paintings (the ratings of regularity and complexity in both sets of stimuli were obtained in the pilot study). In the main experiment, 32 participants rated the aesthetic pleasantness of paintings with congruent, incongruent, and no music background. In addition, they rated the music-paintings matching (how well the music goes with the painting). The results show no effect of congruence on aesthetic pleasantness ratings. The effect on the perceived matching was significant; matching is higher in the congruent compared to the incongruent condition. These findings suggest that congruency has a strong effect on the perceptual aspect of the music-paintings compatibility (visuo-auditory similarity) and no effect on the aesthetic aspect (liking).

## 1. Introduction

Cross-modal correspondence refers to the experience of the similarity of information from different sensory modalities [1,2,3,4]. It is close to the phenomena of synesthesia, but unlike direct sensory experience in synesthesia (e.g., the color of sound is seen and the sound of color is heard), cross-modal correspondence includes “suggested” associations of sensory representations. In his classical example, Sapir [5] noticed that the pseudo-name “Mil” suggests a smaller, and “Mal” a larger object. Similar soundness-size associations mentioned Gombrich [6] in his “Ping-Pong” and “Yin-Yang” examples: “Ping” and “Yin” suggest smaller objects, whereas “Pong” and “Yang” suggest larger object. In the other classical example Köhler [7,8] demonstrated soundness-shape association; the pseudo-name “Takete” suggests an angular pattern, and the pseudo-name “Maluma” a curvilinear pattern. Ramachandran and Hubbard [9] found a similar phonetic symbolism in shapes called “Kiki” (angular shape) and “Buba” (oval shape).

In the present study we focused on the correspondence between two more complex auditory and visual stimuli music and paintings. Previous studies suggested that certain melodies are regularly associated with particular colors: while “sad” music (minor key) dominantly induces associations of blue, “happy” music (major key) is usually associated with yellow [10,11]. Other studies, which investigated the interaction of different styles or genres of music and visual arts (e.g., paintings and architecture) have found that the stylistic congruence between paintings and music increased the aesthetic evaluation [12,13]. For instance, paintings of Kandinsky are preferred while jazz music is playing in the background, whereas William Turner’s paintings are preferred when followed by classical music [14]. Actis-Grosso and collaborators [15] found that figurative paintings, in general, are experienced as more pleasant while followed by classical music in the background, whereas, abstract paintings are experienced as more pleasant when followed by jazz music. Interestingly, this study indicated that, paintings are experienced as more pleasant in conditions with music in comparison to neutral conditions (in silence).

Stimuli that were used in the previous studies were usually classified in broad categories, such as figural and abstract art or classical music and jazz [15]. These categories covered a wide spectrum of diverse and even very distinct styles and sub-genres; compare, for instance, abstract expressionism with geometric abstraction or free jazz with cool jazz. In the present study we attempted to specify this variability more precisely, using two “super-modal” dimensions—regularity and complexity. As a stimulus property, regularity could be defined as a structural articulation or a “good” organization of both paintings (e.g., symmetry) and musical compositions (e.g., harmony), whereas complexity could be specified as a structural heterogeneity, measured by the number of different components within both paintings (e.g., number of colors, shapes, lines, etc.,) and musical compositions (e.g., number of melodic changes, instruments, etc.,). Many empirical studies and theoretical analyses emphasized the crucial roles of regularity and complexity in perception and aesthetic preference [16,17,18,19,20,21,22,23,24,25,26,27,28]. Also, in numerous semantic differential studies, principal components called regularity and complexity were extracted as dimensions of subjective experience of both visual arts [18,29,30] and music [31].

The main purpose of the present study was to evaluate the relevance of regularity and complexity as factors of cross-modal correspondence in a paintings-music matching task. In order to reduce the confounding effect of explicit meanings, we used only abstract paintings and instrumental jazz compositions. Other possible confounding factors, such as familiarity and aesthetic pleasantness were also controlled.

The second aim of this study was to replicate the previous findings that (a) paintings followed by congruent music were preferred compared to an incongruent situation [12,13,15]; and that (b) generally, background music induces higher aesthetic ratings of paintings compared to the “silent” situation [15].

Two experiments were conducted. In the preliminary study, stimuli for the main experiment were selected. In the main experiment participants rated (a) paintings-music correspondence (matching ratings) and (b) the liking of paintings (aesthetic ratings) in different conditions—congruent, incongruent and silent.

## 2. Preliminary Study

The aim of this study was to create the stimulus sets for the main experiment.

### 2.1. Materials and Methods

#### 2.1.1. Participants

Thirty-five undergraduate students from the Department of Psychology participated in the experiment (25 females).

#### 2.1.2. Stimuli

In a pre-selection session three art experts selected 71 abstract paintings and 36 jazz excerpts, so to cover the widest possible range of different styles and sub-genres. Images of paintings were downloaded from Google, while musical compositions were downloaded from the YouTube channel (The Audacity program was used for cropping the 30 s excerpts).

#### 2.1.3. Procedure

Paintings were displayed online via the Qualtrics platform. They were presented in a random order, lasting 5 s. The same sample of participants was exposed to the randomized jazz excerpts via stereo sound system. Participants rated paintings and jazz excerpts on the four 7-step bipolar scales: Irregular-Regular, Simple-Complex, Unpleasant-Pleasant, and Unfamiliar-Familiar.

### 2.2. Results

Thirty-two abstract paintings and eight jazz excerpts were selected. Four sets of both paintings (8 stimuli per set) and jazz excerpts (2 stimuli per set) were specified, so that all sets had similar average ratings on pleasantness and familiarity, but different combinations of regularity/complexity ratings: low regularity–low complexity, low regularity–high complexity, high regularity–low complexity, and high regularity–high complexity (see Table 1 and Table 2).

The analyses of variance have shown the significant effects of paintings sets for regularity, F(3, 31) = 72.59, *p* < 0.001, and complexity F(3, 31) = 86.54, *p* < 0.001. A post hoc test (Bonferroni) indicated significant differences between R+ and R− sets on regularity and C+ and C− sets for complexity (all *p* < 0.001). The effects for pleasantness and familiarity were missed, showing that the sets were equalized by those two variables.

The analyses of variance have shown the significant effects of sets of music excerpts for regularity, F(3, 31) = 23.38, *p* < 0.001, and complexity F(3, 31) = 381.11, *p* < 0.001. A post hoc test (Bonferroni) indicated the significant differences between R+ and R− sets on regularity and C+ and C− sets for complexity (all *p* < 0.001). The effects for pleasantness and familiarity were missed, showing that the sets were equalized by those two variables.

All stimuli are listed in Appendix A (32 paintings) and Appendix B (8 musical compositions).

## 3. Main Experiment

In the main experiment the painting-music correspondence (matching) was investigated. Also, the aim of this experiment was to evaluate the hypothesis that a higher congruence between the paintings and the music should induce a higher aesthetic liking of the paintings.

### 3.1. Materials and Methods

#### 3.1.1. Participants

32 volunteers employed by the “VIP Mobile” company, predominantly from HR, Marketing, and IT sectors (mean age of approximately 29 years; 20 females).

#### 3.1.2. Stimuli

32 abstract paintings and 8 one-minute excerpts of jazz compositions that were classified in four sets specified in the pilot study (see Stimulus section).

#### 3.1.3. Procedure

**Figure 1:**
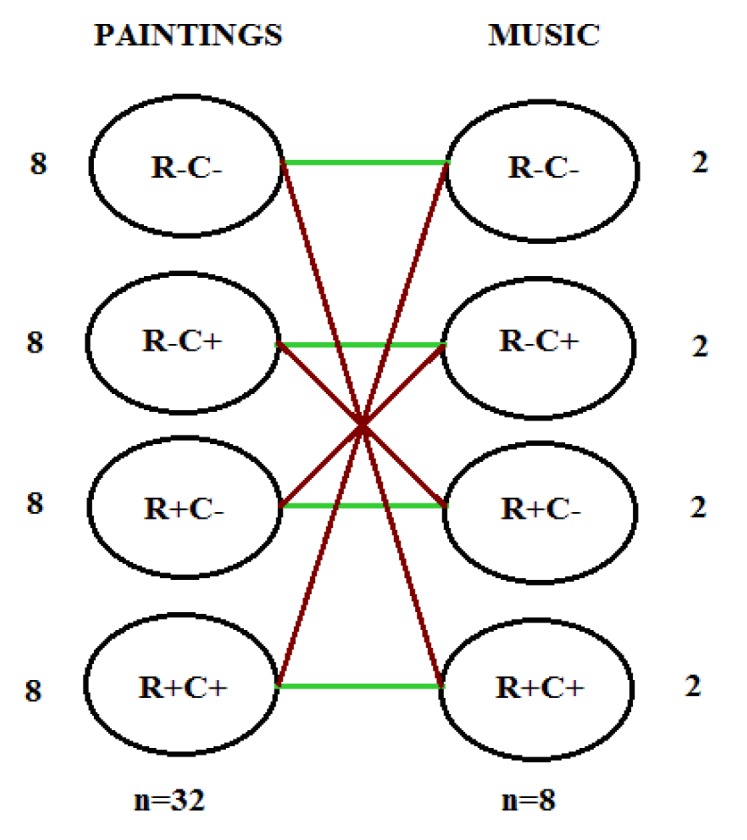
Four groups of visual and music stimuli based on regularity/complexity combination; green (parallel) lines represent congruent conditions, whereas, red (crosswise) lines represent incongruent conditions.

In the first part of the experiment, participants were asked to rate paintings (1) simultaneously presented with congruent music (paintings and music had similar ratings on Regularity and Complexity, i.e., R+C+ and R−C−); and (2) simultaneously presented with incongruent music (i.e., paintings and music had different ratings on regularity and complexity, i.e., R+C− and R−C+). The instruction did not mention music. Namely, participants were asked to rate the paintings, not to evaluate the overall experience of the picture and music. All combinations of stimuli were shown in Figure 1. Four randomized paintings (2 congruent and 2 incongruent with the composition) were presented per one musical excerpt, each in the duration of 5 s. After removing the painting image from the screen, participants rated the liking of the painting, as well as the perceived matching of the music and the painting exposed together (only in paintings-music conditions). Half of the paintings were used in either a congruent (C) or incongruent (I) condition, which means that participants were not observing the same paintings in both conditions. In the second part of experiment participants rated the same paintings in a neutral condition, i.e., in silence (S). The order of the experimental phases with music (C/I) and in silence (S) was counterbalanced between subjects. In addition, the order of C and I conditions within a music phase was counterbalanced as well.

#### 3.1.4. Design

The experiment included three one-factorial designs:

1. Congruence (C/I conditions);
2. Congruence/silence (C/Sc; Sc denotes the paintings that were seen in both C and S conditions);
3. Incongruence/silence (I/Si; Si denotes the half of paintings which were seen in both I and S conditions).

Dependent variables:

1. Ratings of paintings liking.
2. Ratings of paintings-music matching.

### 3.2. Results

#### 3.2.1. Ratings of Paintings-Music Matching: Is the Congruence Defined by Regularity and Complexity between Music and Paintings Perceivable?

**Figure 2:**
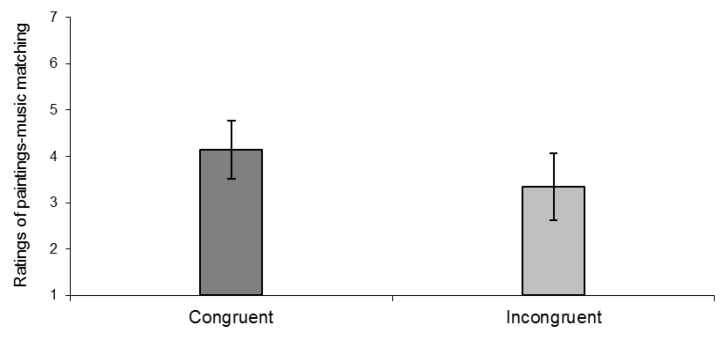
Ratings of paintings-music matching in congruent and incongruent conditions.

Analysis indicates that the ratings of paintings-music matching are, expectedly, significantly higher in congruent condition than in incongruent condition, t(30) = −3.25, *p* < 0.01 (see Figure 2).

#### 3.2.2. The Aesthetic Effect of Congruence: Is the Liking of the Paintings Higher in Congruent Conditions Compared to Incongruent Conditions?

No difference between congruent and incongruent conditions was obtained, t(30) = −0.57, *p* > 0.05.

#### 3.3.3. The Aesthetic Effect of Music Presence: Is the Liking of the Paintings Higher When They Were Followed by Music Background Compared to Silence Conditions?

No difference in preference between music condition and silent condition was obtained, t(31) = 0.7, *p* > 0.05.

## 4. Discussion

The finding that paintings and music are perceived as more corresponding in congruent conditions suggests that (in)congruent situations were specified appropriately in the pilot study. In other words, our results indicate that congruence based on perceptual dimensions such as regularity and complexity could be taken as the basis of evaluation of a paintings-music matching.

On the other hand, our results suggest that music per se has not appeared to be a significant factor in navigating the preference of paintings. One of the possible explanations could be the instruction which the participants were given. By asking them to evaluate only the preference of the painting, they could easily divide the aesthetic experience of two art modalities and focus only on the experience of the paintings while ignoring the experience of the music. Instead of that, further experiments should adopt a more gestaltistic approach and focus on the aesthetic experience of the whole multimodal percept. Apart from the effect of instructions, these results might also be caused by the genre homogeneity of stimuli (abstract paintings and jazz). Namely, it is possible that an increase in the genre variability would induce a stronger expression of the effect of music, similar to that identified in a previous study [15]. In order to test this assumption, additional studies should be conducted. These studies should include different genres, which would increase stimulus variability, as well as a systematic control of the perceptual characteristics, such as regularity and complexity. Although the congruence between two art modalities seems to be a significant aspect when it comes to the cognitive part of aesthetic evaluations (perceived correspondence), it does not show any impact on the evaluative aspect—aesthetic preference. It might be argued that evaluation, a dimension which was controlled across all the stimulus sets, is an important aspect underlying personal aesthetic experience which potentially affects one’s aesthetic preference more than regularity and complexity do.

## Figures and Tables

**Table 1:** The table shows means (M) and standard deviations (SD) of the ratings of four sets of paintings. Sets were specified as combinations of either low regularity (R−) or high regularity (R+) with both low complexity (C−) and high complexity (C+).

Paintings	N	Regularity		Complexity		Pleasantness		Familiarity	
		M	SD	M	SD	M	SD	M	SD
R+C+	8	5.41	0.39	4.76	0.26	4.29	0.26	3.58	0.36
R+C−	8	5.47	0.35	2.36	0.59	4.03	0.19	3.77	0.4
R−C+	8	2.89	0.68	5.42	0.46	4.03	0.32	3.72	0.43
R−C−	8	3.43	0.21	3.39	0.26	4.09	0.36	3.39	0.38

**Table 2:** Table shows means (M) and standard deviations (SD) of the ratings of four sets of music excerpts. The sets were specified as combinations of either high regularity or low regularity (R−) (R+) with both high complexity (C+) and low complexity (C−).

Music	N	Regularity		Complexity		Pleasantness		Familiarity	
		M	SD	M	SD	M	SD	M	SD
R+C+	8	6.02	0.20	5.31	0.18	4.36	0.11	3.53	0.13
R+C−	8	5.92	0.20	2.6	0.02	4.17	0.02	3.4	0.75
R−C+	8	4.34	0.08	3.38	0.13	4.17	0.11	3.28	0.09
R−C−	8	2.31	0.97	6.14	0.07	4.19	0.53	3.25	0.27

## Appendix A. Paintings

Paintings distributed in sets: high (+) and low (−) regularity (R) and complexity (C).

**R−C−**
Henri Michaux—Untitled
Kazimir Malevich—Suprematist Painting (with Black Trapezium and Red Square), 1915
Linda Vachon—Untitled
Paul Klee—Fuge in Rot, 1921
Richard Diabenkorn—Untitled, 1952
Robert Motherwell—Dance I, 1978
Robert Motherwell—Untitled, 1981
Sam Francis—Untitled, 1964
**R−C+**
Alan Crockett—Cross Talk, 2017
Jackson Pollock—The She Wolf, 1943
Jackson Pollock—Composition
Maarten Jansen—Abstract Aberration, 2009
Vojkan Djurdjevic—Bombers, 2013
Wassily Kandinsky—Composition VII, 1913
Wassily Kandinsky—Untitled, 1916
Wassily Kandinsky—In Blue, 1925
**R+C−**
Clare Rojas—Untitled, 2013
Kazimir Malevich—Black Square, 1915
Kenneth Noland—Days and Nights, 2008
Louis Reith—Untitled
Mark Rothko—Black, Red and Black, 1968
Piet Mondrian—Composition with red, blue and yellow, 1929
Sonia Delaunay—Composition, 1934
Zanis Waldheims—Untitled
**R+C+**
Clayton Kashuba—Clashing, 2009
Ella Prakash—Untitled
Frantisek Kupka—The shape of the blue (Tvar Modre), 1913
George Sanen—Conversation with Jackson Pollock No. 42, 2015
Kordula Cagemann—Untitled
Matt W. Moore—Untitled Spraypaint (Exhibition Gravity, Paris, 2012)
Piet Mondrian—Composition in Blue, Gray and Pink, 1913.
Wassily Kandinsky—Reciprocal Accords, 1942

## Appendix B. Musical compositions

Musical compositions distributed in sets: high (+) and low (-) regularity (R) and complexity (C).

**R−C−**
Charlie Parker and Miles Davis—Night in Tunisia, 00:52–01:22
John Coltrane—Equinox, 02:30–03:00
**R−C+**
Charles Mingus—Boogie Stop Shuffle, 02:17–02:25, 03:21–03:43
Weather Report—Havona, 04:09–04:39
**R+C−**
Miles Davies—Blue in green, 03:00–03:30
Yusef Lateef—Love theme from Spartacus, 02:16–02:46
**R+C+**
New York Ska Jazz Ensemble—Professor Bebop, 00:16–00:46
New York Ska Jazz Ensemble—Take Five, 00:12–00:42
